# Vascularization of primary and secondary ossification centres in the human growth plate

**DOI:** 10.1186/s12861-014-0036-7

**Published:** 2014-08-28

**Authors:** Sonja M Walzer, Erdal Cetin, Ruth Grübl-Barabas, Irene Sulzbacher, Beate Rueger, Werner Girsch, Stefan Toegel, Reinhard Windhager, Michael B Fischer

**Affiliations:** 1 Department of Orthopaedics, Karl Chiari Lab for Orthopaedic Biology, Medical University of Vienna, Waehringer Guertel 18-20, Vienna, 1090, Austria; 2 Department of Pathology, Medical University of Vienna, Waehringer Guertel 18-20, Vienna, 1090, Austria; 3 Clinic for Blood Group Serology and Transfusion Medicine, Waeringer Guertel 18-20, Vienna, 1090, Austria; 4 Department of Pediatric Orthopaedics, Orthopaedic Hospital Speising, Speisinger Strasse 109, Vienna, 1130, Austria; 5 Department for Health Sciences and Biomedicine, Danube University Krems, Dr.-Karl-Dorrek-Strasse 30, Krems, 3500, Austria

**Keywords:** Growth plate, vascularisation, Primary ossification centres, Secondary ossification centres, Progenitor cells

## Abstract

**Background:**

The switch from cartilage template to bone during endochondral ossification of the growth plate requires a dynamic and close interaction between cartilage and the developing vasculature. Vascular invasion of the primarily avascular hypertrophic chondrocyte zone brings chondroclasts, osteoblast- and endothelial precursor cells into future centres of ossification.

Vascularization of human growth plates of polydactylic digits was studied by immunohistochemistry, confocal-laser-scanning-microscopy and RT-qPCR using markers specific for endothelial cells CD34 and CD31, smooth muscle cells α-SMA, endothelial progenitor cells CD133, CXCR4, VEGFR-2 and mesenchymal progenitor cells CD90 and CD105. In addition, morphometric analysis was performed to quantify RUNX2^+^ and DLX5^+^ hypertrophic chondrocytes, RANK^+^ chondro- and osteoclasts, and CD133^+^ progenitors in different zones of the growth plate.

**Results:**

New vessels in ossification centres were formed by sprouting of CD34^+^ endothelial cells that did not co-express the mature endothelial cell marker CD31. These immature vessels in the growth plate showed no abluminal coverage with α-SMA^+^ smooth muscle cells, but in their close proximity single CD133^+^ precursor cells were found that did not express VEGFR-2, a marker for endothelial lineage commitment. In periosteum and in the perichondrial groove of Ranvier that harboured CD90^+^/CD105^+^ chondro-progenitors, in contrast, mature vessels were found stabilized by α-SMA^+^ smooth muscle cells.

**Conclusion:**

Vascularization of ossification centres of the growth plate was mediated by sprouting of capillaries coming from the bone collar or by intussusception rather than by de-novo vessel formation involving endothelial progenitor cells. Vascular invasion of the joint anlage was temporally delayed compared to the surrounding joint tissue.

## Background

The growth plate primarily accounts for the longitudinal growth of bone, and is anatomically subdivided into a series of zones with unique morphological and biochemical features [1]-[8]. The resting zone (RZ, germinal layer) is formed by small, uniform, compactly located chondrocytes, rich in lipid and cytoplasmic vacuoles, that occur individually or in pairs and are embedded in the extracellular matrix (ECM). The RZ is characterized by low rates of chondrocytes replication, proteoglycan and collagen type IIB synthesis. In the proliferative zone (PZ) chondrocyte are tightly bound in columns parallel to the axis of the length of the bone where they can proliferate and differentiate. The hypertrophic zone (HZ), with its upper zone of maturation and the lower zone of degeneration, is the layer where blood vessels invade, along with chondroclasts that degrade and remodel the cartilage extracellular matrix (ECM). In the zone of chondrocyte maturation, extracellular matrix consisting of collagen type II, IX and XI, the proteoglycans aggrecan, decorin and biglycan and other non-collagenous proteins such as cartilage oligomeric protein (COMP), which allow the recently divided chondrocytes to separate from each other. Osteoblast precursor cells that adhere to the remnants of the cartilage ECM, form bone tissue in primary ossification centres to assemble the provisional calcification zone (CZ). The progression of chondrocytes from the resting over the proliferating to the hypertrophic state of differentiation that culminates in matrix vesicle calcification happens within hours and is an active process that ends with apoptosis and/or autophagic cell death [7]. An encircling fibrochondrosseous structure surrounding the growth plate called the perichondrial groove of Ranvier (GOR) and the ring of LaCroix (ROL), harbours pre-chondrocytes with a high proliferative capacity responsible for the circumferential growth of cartilage and a developed vascularisation [8]-[11].

Vascular invasion of the primarily avascular hypertrophic chondrocyte zone of the growth plate is a prerequisite for the process of endochondral bone formation and occurs in a sequence of events [12]-[15]. There is no evidence of in vivo one step trans-differentiation of chondrocytes to osteocytes. This switch requires a dynamic and close interaction between the cartilage and vascular structures, involving two types of molecules in this process: proteases and growth factors such as metalloproteinase 9 (MMP9), gelatinase B (GelB), vascular endothelial growth factor (VEGF), fibroblast growth factor 18 (FGF18) [13],[16]-[18]. Endothelial cells coming from the bone collar invade the terminal layer of apoptotic chondrocytes to form vascular channels [19]. These newly formed blood vessels provide access for several highly specialized progenitor cells involved in the initiation and regulation of osteogenesis, such as chondroclasts-, osteoblast-, and osteoclast-progenitors, as well as endothelial- and pericyte (smooth muscle) progenitor cells inducing vasculogenesis [13],[14],[19]. Collectively, chondroclasts that differentiate from chrondroclast-precursors degrade and remodel the cartilage extracellular matrix (ECM), so osteoblast precursor cells can adhere to the remnants of the cartilage ECM, differentiate to osteoblasts and form bone tissue in primary ossification centres [20]. The ECM deposited by RUNX2/3 driven hypertrophic chondrocytes serves as a template for subsequent bone formation and these cells can also secrete proteins such as receptor activator of NF-κB ligand (RANKL), Indian hedgehog (Ihh) and VEGF that control the activity of osteoblast-, osteoclast- and endothelial precursor cells coming in from the circulation during the process of endochondral ossification [16]-[18],[21]-[26].

In order to investigate new vessel formation in primary- (POCs) and secondary ossification centres (SOCs) of the developing human growth plate, immunohistochemistry, confocal laser scanning microscopy and RT-qPCR were used with markers expressed on endothelial cells (CD34 and CD31), on hematopoietic precursor cells (CD133 and CXCR4), on smooth muscle cells (α-SMA), and on mesenchymal precursor cells (CD90 and CD105) [8]-[11],[27]. In addition, morphometric analysis was applied to quantify the occurrence of the respective cells in the growth plate and in the surrounding perichondrial area.

## Results

### Terminally differentiated chondrocytes express transcription factors RUNX2 and DLX5 and chondroclasts express RANK in newly formed ossification centres

**Figure 1:**
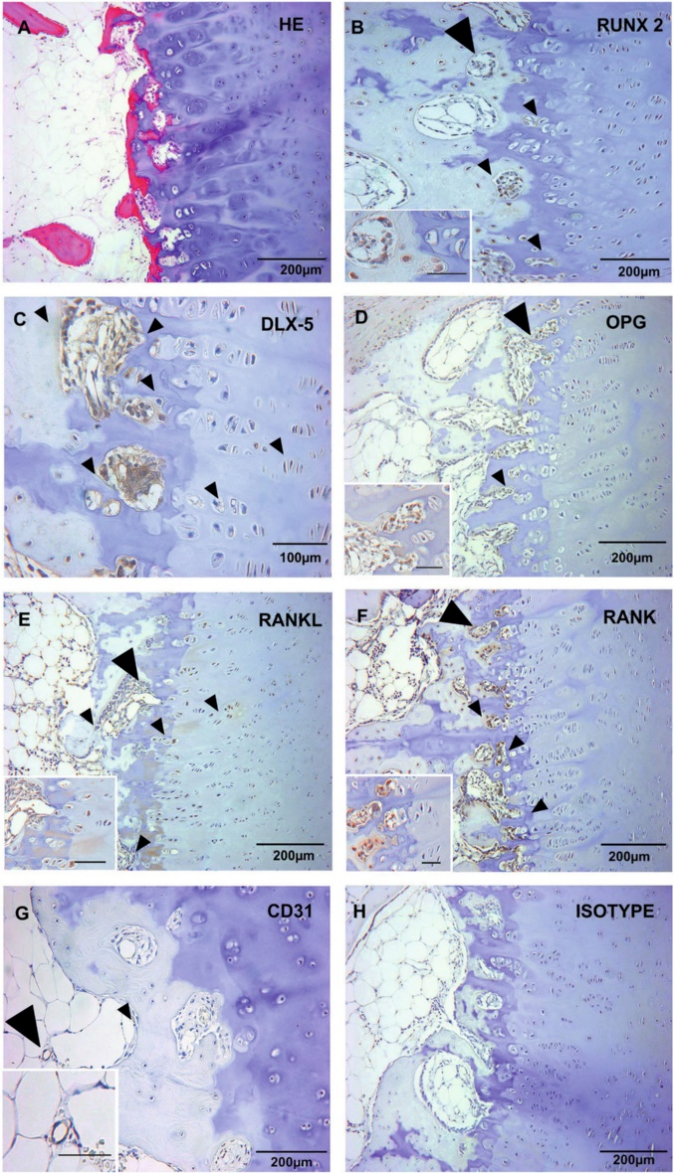
**RUNX-2, DLX-5, OPG, RANKL, RANK and CD31 expression in growth plate (n = 23).** Longitudinal sections of the three investigated histological zones defined as calcification zone (CZ), hypertrophic zone (HZ) and proliferating zone (PZ) were stained with hematoxilin and eosin (HE) **(A)** and Immunhistochemical staining with specific mABs for RUNX-2 **(B)**, DLX-5 **(C)**, OPG **(D)**, RANKL **(E)**, RANK **(F)** were shown in one representative sample including one representative isotype control **(H)**. Large arrowheads indicate the area of the insert (*scale bar* equals 50 μm), small arrowheads indicate specific cells. Hypertrophic chondrocytes expressed RUNX-2 **(B)** and DLX-5 **(C)**. Multinucleated RANK^+^ chondroclasts with brush borders were found in the CZ **(F)**, and vessels in newly formed bone but not in POCs expressed CD31 the marker for mature endothelial cells **(G)**.

**Figure 2:**
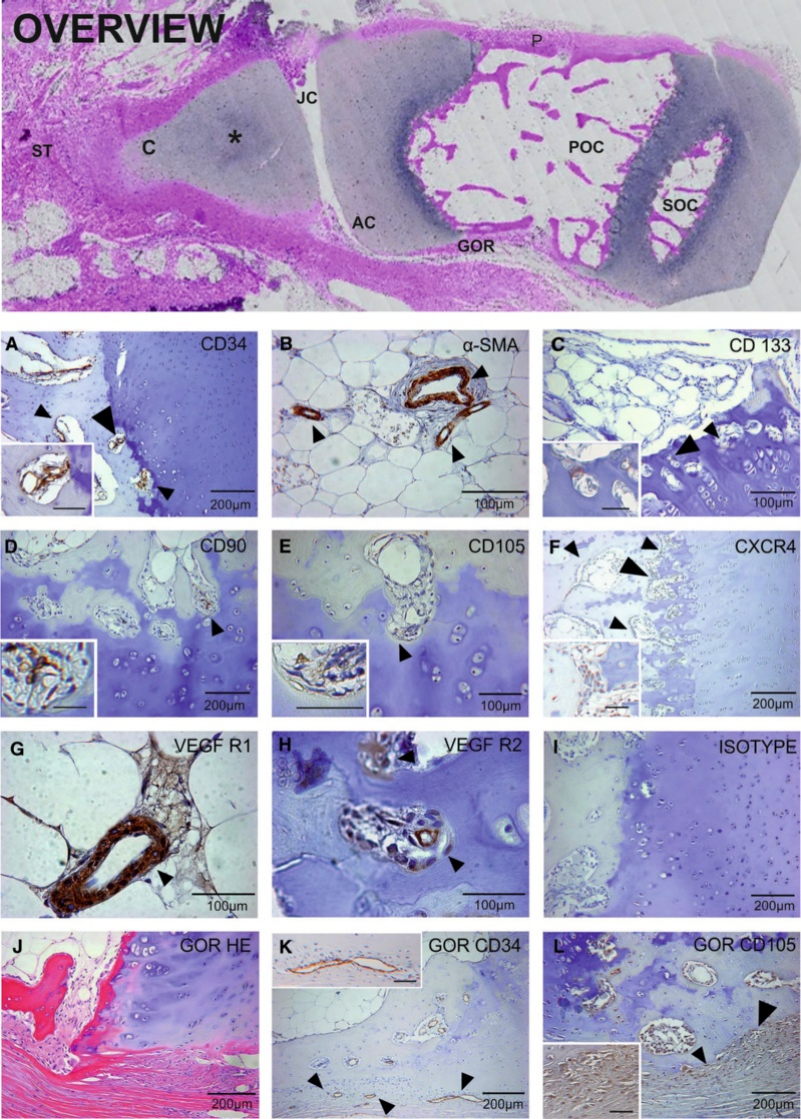
**CD34, α-SMA, CD133, CD90, CD105, CXCR4, VEGFR1, VEGFR2 expression in growth plate and in the surrounding GOR (n = 23).** An entire digit section was scanned by TissueFAXS (overview) including uncalcified cartilage (C), area of ongoing calcification (*), joint cavity (JC), surrounding soft tissue (ST), articular cartilage (AC), Groove of Ranvier (GOR), POC, and SOC. Immature endothelial cells in POCs expressed CD34 **(A)**, abluminal coverage with α-SMA^+^ smooth muscle cells was found on vessels in newly formed bone but not in POCs and SOCs **(B)**. Single CD133 precursor cells were found in POCs in close proximity to newly formed vessels **(C)**, mesenchymal progenitor cells expressing CD90 **(D)** and CD105 **(E)** co-localized with CD34^+^ endothelial cells in POCs. CXCR4+ cells **(F)** could be localized in POCs. VEGFR1 **(G)** is expressed on endothelial cells of vessels in newly formed bone, while VEGFR2 **(H)** is expressed on immature CD34^+^ endothelial cells in POCs. The groove of Ranvier (GOR) **(J)** showed intense vascularisation **(K)** with mesenchymal progenitors expressing CD105 **(L)**. A representative isotype control was included **(I)**. Large arrowheads indicate the area of the insert (*scale bar* equals 50 μm), small arrowheads indicate specific cells.

Of the 23 patients included in this study, digits of 15 patients had well developed growth plates with primary and secondary ossification centres (Table 1, Figures 1 and 2) while the remaining eight showed developing as well as undeveloped growth plates with cartilage anlagen (Table 1). We found hypertrophic chondrocytes located in the PZ that started to express the transcription factors RUNX2 and DLX5 in digits with active endochondral ossification of the growth plate (Figure 1B,C). A maximum of RUNX2 and DLX5 expression was found in terminally differentiated chondrocytes of the HZ (Figure 1B,C). Layers of multiple hypertrophic chondrocytes located in the PZ and certain terminally differentiated chondrocytes of the HZ also expressed RANKL in a developmental stage before they underwent apoptosis and/or autophagic cell death (Figure 1E). In POCs and SOCs that assembled the provisional CZ, multinucleated chondroclasts expressing RANK on their surface showed ruffled border formation to degrade and resorb the cartilage matrix, while the osteoblasts in newly formed bone expressed OPG and RANKL (Figure 1D,E,F). The remaining eight fingers, where the growth plate had not yet fully developed, presented either an intact cartilage anlage or showed initiation of a primary centre of ossification in the middle of the cartilage model. These newly formed ossification centres harboured hypertrophic chondrocytes expressing RUNX2, DLX5 and RANKL, multinucleated osteoclasts with ruffled border formation along the lacunae expressing RANK on their surface and osteoblast expressing RANKL and OPG according to the extent of endochondral ossification (data not shown).

**Table 1:** Description of the different developing stages of growth plate samples

**Patient**	**Sex**	**Age range**	**Stage of development**			
	**F: female; M: male**	**months at date of surgery**	**Developing stage**	**Endochondral ossification**	**GOR**	**ROL**
1	F	12	+++	++	+++	+++
2	F	11	++	+	+++	+++
3	F	11	+++	+++	+++	+++
4	F	54	+	o	o	o
5	M	54	+++	+++	+++	+++
6	F	26	+	+	o	o
7	F	11	+++	+++	+++	+++
8	M	18	++	o	o	o
9	M	11	++	o	o	o
10	M	26	+++	+++	+++	+++
11	M	6	++	+	+++	o
12	M	11	+++	+++	+++	+++
13	M	16	+++	+++	+++	+++
14	M	41	++	++	+	+++
15	F	105	+++	+++	+++	+
16	F	13	++	++	+++	+++
17	M	33	+++	+++	+++	+++
18	F	7	+++	++	+++	+++
19	M	11	+++	++	+++	+++
20	F	25	+++	+++	+++	+++
21	M	45	+++	+++	+++	+++
22	F	46	+++	++	+++	+++
23	M	7	+++	++	+++	+++

o not developed; + beginning to develop; ++ developing; +++ developed.

### New vessel formation in primary and secondary ossification centres is mediated by sprouting rather than by de-novo vessel formation

**Figure 3:**
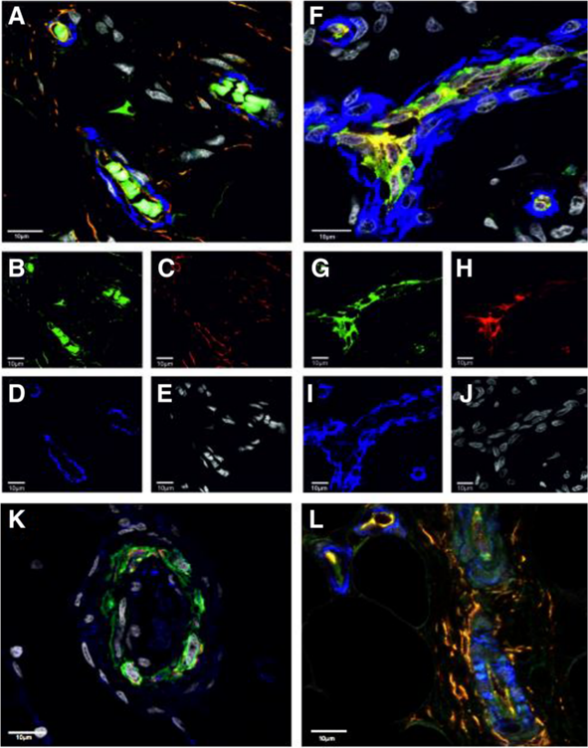
**A-E: Spouting of new vessels in primary ossification centres of the growth plate.** CD34^+^ (green) endothelial cells formed cascades that had not been stabilized by α-SMA bearing sub-endothelial smooth muscle cells (blue) in immature vessel formations. **F**-**J**: Vasculature in the surrounding articular soft tissue showed continuous lining of CD34^+^ (green) endothelial cells partly sharing (yellow) endothelial CD31^+^ (red) presence in already α-SMA bearing sub- endothelial smooth muscle cells (blue) stabilized mature vessels. **K**-**L**: Mature vessels with a lumen and CD34^+^ (green) and CD31^+^ (red) endothelial cells as well as α-SMA bearing smooth muscle cells (blue) A-L were displayed in confocal Laser Scanning Microscopy (630x magnification), all cellular nuclei were counterstained with DAPI (white). To enable comparison across the panel of CD34, CD31 and α-SMA, parameters for data acquisition were kept constant.

In POCs and SOCs of the provisional CZ, CD34^+^ endothelial cells were shown to form vascular arcades and immature capillaries that had not yet been stabilized by α-SMA bearing subendothelial smooth muscle cells (Figure 2A). Precursor cells from the mesenchymal lineage expressing CD90 and CD105 co-localize with CD34^+^ immature endothelial cells of the newly formed sprouts (Figure 2A,D,E). Only a coordinated and finely tuned concurrent development of immature CD34^+^ endothelial cells and subendothelial CD90^+^/CD105^+^ smooth muscle cell precursors is a prerequisite for successful new vessel formation. In close proximity to these immature vessels, precursor cells expressing CD133 could be found in POCs but not in SOCs (Figure 2C). These very rarely occurring cells are most likely bone marrow derived and enter the POCs from the circulation during the process of vascular invasion. They were shown to express the chemokine receptor CXCR4 (Figure 2F), the receptor for CXCL12 (SDF-1) involved in selective home of precursor cells to sites of new vessel formation [28]. In accordance with previous studies CXCR4 is widely expressed on precursor cells of the endothelial lineage, osteoblasts, RANKL activated osteoclasts and activated lymphocytes [29],[30]. In addition to their appearance in the POCs, CD133^+^ precursor cells could be found on the surface of the articular cartilage where they formed a thin layer of cells, as well as in the perichondrium and periosteum (data not shown). Of the two receptors binding to the different isoforms of VEGF, VEGFR1 was found to be expressed by mature endothelial cells in vessels of newly formed bone and VEGFR2 on immature endothelial cells in vascular arcades and developing vessels within POCs (Figure 2G,H). We found no evidence of VEGFR2^+^ expression on CD133^+^ endothelial precursor cells in newly formed vessels within POCs. This rare population of CD133^+^VEGFR2^+^ endothelial precursors were shown to represent less than 2% of stem cells in the circulation and had a high potential to differentiate along the endothelial lineage *ex vivo*[31],[32]. During their maturation to endothelial cells, they lose CD133 and gain CD31 and vWF [33]. Angiogenesis with sprouting of new capillaries from pre-existing vessels and intussusception, with division of one vessel into two by the formation of a new vessel wall in the lumen of the original vessel, is more likely responsible for new vessel formation than vasculogenesis mediated by CD133^+^VEGFR2^+^ endothelial precursors [33]. In the newly formed bone, in contrast, the majority of CD34^+^ endothelial cells were also CD31^+^ and vessels were covered with α-SMA^+^ smooth muscle cells (Figure 2B), indicating that these vessels were mature and stabilized (Figure 1G, Figure 2B and see for more detail Figure 3K,L). Digits where the removal of embryonic cartilage by endochondral ossification had not yet been initiated showed no occurrence of CD133^+^ precursor cells, providing further evidence that new vessel formation during the process of vascular invasion of the cartilage starting from the periosteal bone collar is mediated by sprouting (data not shown).

### Vascularisation of the groove of ranvier and the ring of LaCroix

Within the perichondrial GOR and ROL we found stabilized vessels with CD34^+^ endothelial cells and abluminal coverage with α-SMA^+^ smooth muscle cells but no evidence for CD133^+^ precursor cells (Figure 2K). The majority of cells resident in the GOR were of mesenchymal origin because they expressed markers for mesenchymal progenitor cells such as CD90 (data not shown) and CD105 (Figure 2L) and lacked CD45 excluding their myeloid origin. The perichondrium and periosteum surrounding the growth plate were highly vascularized with stabilized vessels and capillaries and harboured CD133^+^ progenitor cells (data not shown). Digits with only one cartilage anlage or those at the beginning of central ossification within the cartilage anlage showed no GOR and ROL comparable to that observed in growth plates (data not shown).

### Morphometric analysis of precursor cells in different areas of the growth plate

**Figure 4:**
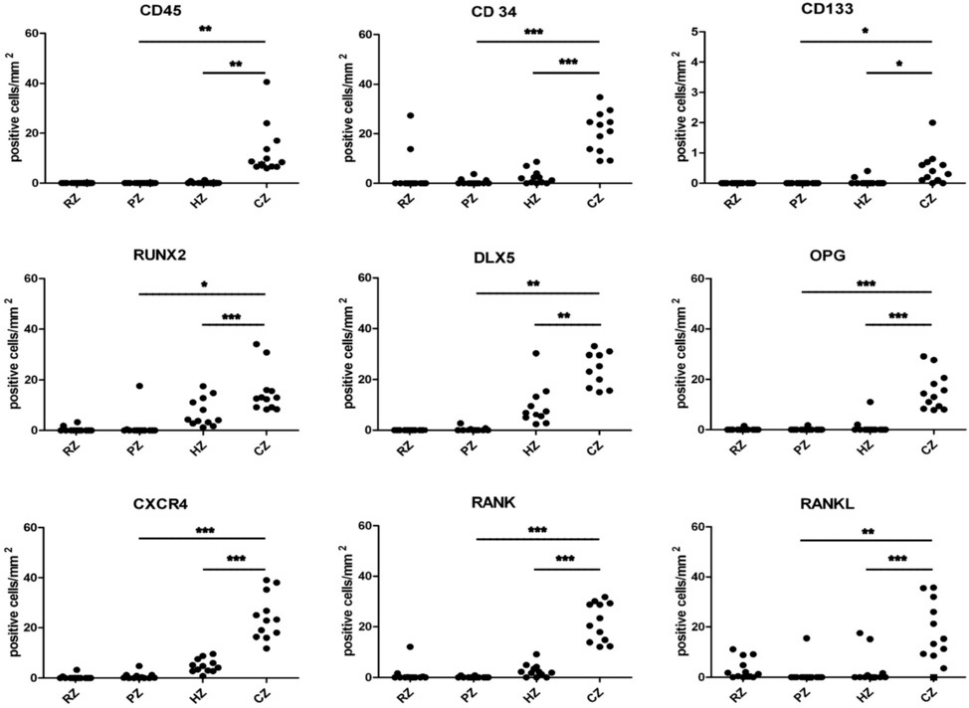
**Morphometric analysis: Number of CD34, CD45, CD133, CXCR4, RANK, RANKL, OPG, RUNX-2 and DLX-5 positive cells/mm**^**2**^**in the resting zone (RZ), proliferating zone (PZ), hypertrophic zone (HZ) and calcification zone (CZ) of the growth plate were counted in 10 fields of 1 mm**^**2**^**(n = 12).** Statistically significant differences are indicated as *p < 0.05, **p < 0.005, ***p < 0.001.

Quantitative histomorphometric measurements giving the numbers of positive precursor cells, hypertrophic chondrocytes and mature endothelial cells found per mm^2^ in the different zones of the growth plate and are depicted in Figure 4.

### Sprouting of new vessels in ossification centres

Laser confocal microscopy was used to further characterize sprouting of new vessels in primary ossification centres of the growth plate in polydactylic fingers. In ossification centres of the provisional CZ, CD34^+^ endothelial cells (green) were shown to form vascular arcades as well as immature capillaries that had not yet been stabilized by α-SMA bearing sub-endothelial smooth muscle cells (blue) (Figure 3A-E). Vascular development within the soft tissue surrounding the growth plate seemed to have proceeded much further than within the provisional CZ of the growth plate. The vasculature within the soft tissue of the joint showed continuous lining of endothelial cells stained with CD34 (green) and partial staining of these endothelial cells with CD31 (red) (Figure 3F-J). Furthermore, stabilization with α-SMA bearing sub-endothelial smooth muscle cells (blue) already occurred, indicating stable mature vessels. The endothelial lining in provisional CZ of the growth plate, in contrast, showed no signs of continuity as depicted by CD34 staining of endothelial cells (green). Several CD34^+^ endothelial cell clusters of 2–4 cells were observed separated from each other with no tendency to connect for alignment. In addition, few CD31^+^ mature endothelial cells (red) were found in provisional CZ and no signs of stabilization of vessels with α-SMA bearing sub-endothelial smooth muscle cells (blue) indicating that these newly formed vessels were immature.

### RT-qPCR of CD34, CD31 and CD133 expression in cultured adherent cells of the growth plate

**Figure 5:**
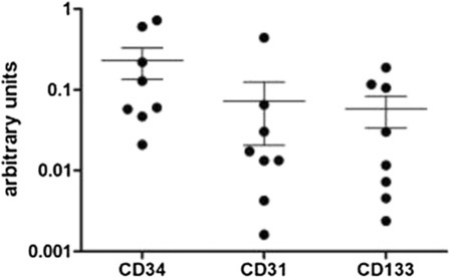
**mRNA levels of CD 34, CD31 and CD133 were quantified (n = 8) using RT-qPCR and expressed as numbers of molecules by 1,000 molecules of GAPDH (NMby1000GAPDH).** Adherent cells isolated from dissected growth plates were cultured and analyzed separately. Results are given as mean values with standard deviation.

Cultured adherent cells of the dissected growth plate were investigated for their expression of CD34, CD31 and CD133 message by RT-qPCR in order to estimate activity of new vessel formation [34]. With respect to GAPDH arbitrarily set at 1,000, the mRNA levels of CD34, CD31, and CD133 were ranging from 0.02 to 0.7, 0.001 to 0.4, and, 0.002 to 0.1, respectively, across all eight independent samples (Figure 5). In comparison, CD34 mRNA tended to be higher expressed than CD31 mRNA in each patient-derived cell population, barely missing the level of significance (p = 0.07).

## Discussion

During the process of endochondral bone formation that leads to the establishment of the diaphyseal POCs and epiphyseal SOCs, a coordinated transition from proliferation to terminal differentiation and hypertrophy of chondrocytes occurs that ends with hypertrophic chondrocytes mineralising their ECM and undergoing apoptosis or autophagic cell death [2],[4],[7]. Endothelial cell invasion starts with the generation of vascularized cartilage canals from the perichondrium into the terminal layer of apoptotic chondrocytes ending with the formation of new capillaries in POCs [19]. In ossification centres, the vascularization was shown to occur prior to hypertrophy, matrix mineralization and subsequent apoptosis or autophagic cell death of chondrocytes [19]. To create a suitable environment for vascular invasion, we found coordinated transition from proliferation to terminal differentiation and hypertrophy of chondrocytes, tightly regulated under the control of transcription factors RUNX2 and DLX5, in human growth plates investigated. Newly formed blood vessels provide access for a myriad of different precursor cells with high specialization involved in the development of ossification centres [9]-[11],[35]. We found evidence for the appearance of CD90^+^ progenitor cells of the mesenchymal lineage in POC and SOC, but progenitor cells of the myeloid lineage expressing CD133 were extremely rare. In POCs but not in in SOCs, single CD133^+^ progenitor cells could be detected next to immature vessels and vascular arcades with a lining of CD34^+^ endothelial cells. Interestingly, CD90^+^ precursor cells from the mesenchymal lineage tend to seek physical contact to CD34^+^ immature endothelial cells of the newly formed endothelial sprouts. These immature vessels lacked abluminal coverage with α-SMA^+^ smooth muscle cells that are important for vessel stabilization because they prevent vessel pruning [36]. It seems likely that only a coordinated and finely tuned concurrent development of immature CD34^+^ endothelial cells and subendothelial CD90^+^ smooth muscle cell precursors is a prerequisite for successful new vessel formation in POCs as well as SOCs. Subendothelial smooth muscle cells can develop from pericytes. These cells can be recruited from neighbouring resident mesenchymal cells through replication, migration and differentiation of other pericytes of the growing vascular bud or they can arise from mesenchymal progenitors [35],[37]. There is a physiological need for cross-differentiation of connective tissue cells in neovascularization, namely the need to adapt different tissues that reside next to one another during the process of vessel stabilization [37]. Although extensively investigated, we found no evidence for the occurrence of endothelial progenitor cells expressing CD133 and VEGFR-2 in POCs and SOCs. It seems likely that new vessels were formed by the sprouting of new capillaries from the pre-existing vessels coming from the bone collar or by intussusception rather than by de-novo vessel formation by endothelial progenitor cells expressing CD133 and VEGFR-2. Recently it was shown that endochondral ossification is required for haematopoietic stem-cell niche formation [36]. Matrigel embedded progenitor cells with surface markers CD105^+^Thy1^−^ (CD90) sorted from 15.5 days post-coitum fetal bones could recruit, when transplanted under the adult mouse kidney capsule, host-derived blood vessels, produce donor derived ectopic bone through a cartilage intermediate and generate a marrow cavity populated by host-derived long-term reconstituting HSCs [36]. These results might support our hypothesis that the CD133^+^ progenitor cells found in POCs and SOCs of polydactylic digits are involved in the formation of future stem-cell niches rather than in vasculogenesis.

Vascularization in GOR and ROL, that serve as a reservoir for pre-cartilaginous cells in the germ layer [38] and in case of ROL keeps the cells from oozing out under axial loading, was found to be more developed than in the corresponding ossification centres. An intact perichondrial zone with optimal vascularization is important for longitudinal bone growth, as Salter-Harris type IV fractures within the GOR led to severe growth disturbances [39],[40]. Small capillaries with CD34^+^ endothelial cells stabilized with α-SMA^+^ smooth muscle cells were found from the onset of GOR and ROL. The GOR was shown to maintain their progenitor properties very similar to bone marrow stem cell niches and harbours an extensive vascular network [9],[10]. Precursor cells within the perichondrial GOR of female New Zealand white rabbits expressed STRO-1, Jagged1 and bone morphogenetic protein receptor 1a (BMPr1a) and had a morphology similar to mesenchymal stem cells. We found progenitor cells of the mesenchymal lineage expressing CD90 and to a certain extent CD105 in the basal layer of the GOR according to previous reports [9]-[11],[35]. When these mesenchymal progenitor cells differentiate, they lose expression of CD90 and CD105 and move to the apical part of the GOR to be dispersed later throughout the cartilage. In a mouse model, these extensive proliferating cells were shown to incorporate BrdU into their DNA after 10–12 days of BrdU feeding, and transfected cells from the GOR initially migrated back to the perichondrial groove and later deeper into the epiphysis [41] or to the surface of the articular cartilage [9]. In addition, the perichondrium and periosteum, adjacent to the physis, was found to be highly vascularized and harboured multiple single CD133^+^ progenitor cells.

Eight of the immature growth plates investigated showed either a cartilage anlage or the beginning of central ossification. The cartilage anlage that forms as a result of mesenchymal condensation and subsequent differentiation into cartilage, is a primary avascular structure, which relies on receiving oxygen and nutrients via diffusion [42]. In this stage of growth plate development, oxygen debt due to a lack of blood vessels was shown to increase the anabolic metabolism of growth plate chondrocytes and up-regulated hypoxia-inducible factors (HIFs) [11],[43]. HIF-1α is necessary for chondrocyte survival during hypoxia, stimulates production of collagen type II, and induces the expression of different VEGF isoforms in chondrocytes. VEGF is first present in the cartilaginous ECM and, upon degradation of this ECM by MMPs like MMP-2, MMP-9 and MMP13, VEGF is released and can bind to VEGFR1 and 2 on vascular endothelial cells, and pre-osteoblasts, thereby favouring vascular invasion and cartilage replacement by bone [44]. In this stage of bone development we found no evidence for VEGFR1 or VEGFR2 expressing endothelial cells or endothelial progenitor cells.

## Conclusions

In conclusion we can say that new vessel formation in the human growth plate is mediated by sprouting of capillaries coming in from the bone collar rather than by *de novo* vessel formation mediated by endothelial precursor cells. Understanding the complex physiology of growth plate development, where a single cell type, the chondrocyte, can render the impulse for the growth of massive bone from embryonic life to adulthood, supported by the growing vascular network, will help to establish new strategies in personalized regenerative medicine.

## Methods

### Patients

Digits of 23 infants (11 females, 12 males) with bilateral polydactyly were collected after surgical removal of the digits between 6 and 105 months after birth. The patients’ tissues were included in the study with informed consent of the parents. The study was performed according to the guidelines of the Medical University of Vienna [45] and the study was approved by the Ethics Commitee of the Medical University Vienna (EK Nr.: 1830/2012).

### Immunohistochemistry and immune-morphometric analysis

The resected polydactylic digits were fixed in 10% formalin for 3 days and then embedded in paraffin after decalcification as described before [45]. After de-paraffination through xylene and graded alcohol, the specimens were cut into slices of approximately 4–6 μm. For removal of endogenous peroxidase the material was treated with 3% H_2_O_2_, followed by antigen retrieval in the microwave (10 min, 150 W) in citrate buffer (pH = 6.0) as previously described [45]. Sections of paraffin embedded tissues were treated with horse serum (CD133, CD34, CD45, CXCR4, RANK and OPG) or goat serum albumin (RANKL, DLX5, RUNX2) for 30 min at room temperature and subsequently incubated with the primary antibodies against CD133 (2.5 μg/ml mouse monoclonal antibody (mAb), Miltenyi Biotec, Bergisch Gladbach, Germany; 1.3 μg/ml goat polyclonal antibody (pAb), Santa Cruz Biotechnology, Santa Cruz, CA), CD34 (2.5 μg/ml mouse mAb, Becton Dickinson, San Jose, CA, or 0.5 μg/ml mouse mAb, Immunotech, Marseilles, France), CXCR4 (2.5 μg/ml mouse mAb, PharMingen, San Diego, CA), VEGFR-2 (2.5 μg/ml mouse mAb, Santa Cruz Biotechnology, Cell Signalling Technology, Danvers, MA), CD45 (3.5 μg/ml mouse mAb, Becton Dickinson), CD90 (2 μg/ml mouse mAb, Novus Biologicals, LLC, Littleton, CO), CD105 (2 μg/ml mouse mAb, Labvision/Neomarkers, Freemont, CA), RANKL (2 μg/ml rabbit mAb, Santa Cruz Biotechnology), RANK (2 μg/ml goat pAb, Santa Cruz Biotechnology), RUNX2 (2 μg/ml rabbit mAb, Oncogene, Cambridge, MA), DLX5 (2 μg/ml rabbit mAb, Chemicon, Billerica, MA), and OPG (2 μg/ml mouse mAb, R&D, Minneapolis, MN) at 4°C overnight. Sections were washed thereafter 3 times in PBS and the reactivity of the primary antibodies was revealed using biotinylated anti-goat IgG (10 μg/ml, Vector Laboratories, Burlingame, CA) for CD133 and RANK, a biotinylated anti-mouse IgG (10 μg/ml, Vector Laboratories) for CD34, CD133, CD45, OPG, VEGFR-2, CD90, CD105, and CXCR4 and a biotinylated anti-rabbit IgG (10 μg/ml, Vector Laboratories) for DLX5, RUNX2 and RANKL for 30 min at room temperature. The unbound secondary antibody was removed by washing three times in PBS, and visualization of antibody staining was achieved using Vectastain ABC (Vector Laboratories, Burlingame, CA) and DAB (Santa Cruz Biotechnology). Isotype controls were included in the protocol (Figures 1H and 2E). Entire digit sections (n = 3) stained by HE were scanned with a Zeiss Observer. Z1 microscope equipped with TissueFAXS and images were analyzed by HistoQuest software (both from Tissue Gnostics GmbH, Vienna, Austria). Morphometric analyses were performed by counting the number of CD45, CD34, CD133, RUNX2, DLX5, OPG, CXCR4, RANK, or RANKL positive cells in 10 fields of 1 mm^2^, selected at random as described earlier by two independent investigators [27].

### Immunofluorescence and confocal laser microscopy

For immunofluorescence, paraffin embedded 2–3 μm tissue sections (n = 10) were deparaffinized and blocked with 10% donkey serum (Jackson Immunotech) for 30 min and subsequently incubated overnight at 4°C with the mouse anti-CD31 (2.5 μg/ml, Dako) in 0.05 M Tris Buffer. After washing the slides with TBS two times, Alexa Fluor (AF) 555 conjugated donkey anti-mouse Ab (2 μg/ml, Life Technologies Invitrogen) was applied for one hour at room temperature (RT) followed by blocking the sections with 20% mouse serum (Jackson Immuno Research Laboratories, West Grove, PA, USA) for 30 min at RT. Sections were blocked with affinity purified Fab fragment donkey anti-mouse IgG (50 μg/ml, Jackson) in TBS/5% donkey for 1 h at RT and with 10% donkey serum (Jackson) for 30 min. to prevent cross reaction. Thereafter, slides were incubated with an AF 488 conjugated CD 34 mAb (2.5 μg/ml, Becton Dickinson, NJ, USA) overnight at 4°C. Repeat blocking of the sections with 20% mouse serum (Jackson Immuno Research Laboratories, West Grove, PA, USA), washing and incubated again with affinity purified Fab fragment donkey anti-mouse IgG in TBS/5% donkey for 1 h at RT to prevent secondary antibody cross reaction with the third primary mouse monoclonal antibody applied. After additional blocking with 10% donkey serum the slides were incubated with an α-SMA mAb (2.5 μg/ml, Sigma-Aldrich, MO, USA) at 4°C overnight and α-SMA staining was visualized using a AF 647 donkey anti-mouse Ab (2 μg/ml, Invitrogen, CA, USA). Nuclei were stained with 1.5 μM 4′,6 – diamino-2-phenylindole (DAPI, Sigma-Aldrich, MO, USA). Isotype controls were included in the protocol (data not shown). Images were acquired with Zeiss LSM 700 confocal laser microscope (63× objective, ZEN black imaging software).

### Cell culture and RT-qPCR

Growth plates (n = 8) were separated under stereo-microscopic control from surrounding soft tissue of the joint. Growth plate cartilage was then manually minced and the remaining soft tissue removed prior to enzymatic digestion at 37°C for 10–18 hours under constant agitation using 2 mg/ml of Collagenase B (Roche Diagnostics, Austria) [11]. Cells were centrifuged at 1300 rpm for 7 min and washed twice with PBS and strained through 40 μm filters (Falcon). Collected cells were plated in alpha-modified minimum essential medium (alpha-MEM; Sigma- Aldrich) supplemented with 10% FBS, 2% Penicillin-Streptomycine (GIBCO Invitrogen, CA, USA), 0.5% L-Glutamine (GIBCO Invitrogen, CA, USA), 0.2% Amphotericine B (PAA Laboratory, Austria), in 6 well plates in a humidified atmosphere with 5% CO2 at 37°C. Medium was changed after 24 h in order to remove non-adherent cells from the culture and thereafter the medium was replaced every 3–4 days until cell density reached 80-90% confluence. Total RNA was extracted from cultured cells of the growth plate using NucleoSpin® RNA (Machery-Nagel, Germany) following the manufacturer’s instructions. Each sample was run on the Agilent 2100 Bioanalyzer Nano LabChip for quality control and quantification of total RNA prior to reverse transcription. RNA integrity numbers were between 9.3 and 10. The purified RNA (10 ng/μl) was reverse transcribed with the High Capacity cDNA Reverse Transcription Kit (Life Technologies, Grand Island, NY, USA) under the following conditions: 25°C for 10 min, 37°C for 120 min followed by 85°C for 5 min. The qPCR reactions were performed on an Applied Biosystems StepOnePlusTM PCR device using SYBR® Green PCR Master Mix (Life Technologies, Grand Island, NY, USA), sequence specific primers for GAPDH, CD34, CD 31 and CD133 at a concentration of 100nM and 1 μl cDNA (diluted 1:5) under the following conditions: 95°C for 10 min followed by 40 cycles of 15 s of denaturation at 95°C, 60 s of annealing (55°C) and 60 s of elongation at 72°C. A melting curve analysis was performed after each run to confirm product specificity. mRNA expression levels were calculated as relative copy numbers considering actual amplification efficiencies and with respect to that of glyceraldehyde-3- phosphate dehydrogenase (GAPDH) set at 1,000. Technically, the protocol deliberately followed the minimal guidelines for the design and documentation of qPCR experiments (MIQE) as recently outlined [34],[46]. A qPCR checklist listing all relevant information is provided to assess the technical adequacy of the used qPCR protocols (Additional file 1).

### Statistic analysis

Paired Student’s t test was used to evaluate differences in the number of CD45, CD34, CD133, RUNX2, DLX5, OPG, CXCR4, RANK, and RANKL positive cells/mm^2^ in the RZ, PZ, HZ and CZ using the SSPS 22.0 and Graph Pad Prism 5 software. Differences were statistically significant when p < 0.05.

## Competing interests

The authors declare that they have no competing interests.

## Authors’ contributions

SMW carried out the immunofluorescence experiments as well as the data analysis, participated in the design of the study, and drafted the manuscript. EC participated in tissue collection, the design of the study and the writing of the manuscript. ST designed the qPCR assays, analyzed and interpreted the qPCR data and participated in drafting the manuscript. RG and BR carried out the histological experiments and contributed to the manuscript. IS participated in the analysis and interpretation of the histological data and contributed to the manuscript. WG and RW included the patients and critically revised the manuscript. MBF conceived, supervised and designed the study and drafted the manuscript. All authors read and approved the final manuscript.

## Additional file

## Supplementary Material

Additional file 1: RT-qPCR checklist with all relevant technical information of RT-qPCR protocols used in the present study. Click here for file
